# Incidentally Discovered Pulmonary Hamartoma: A Case Report and Review of the Literature

**DOI:** 10.7759/cureus.104563

**Published:** 2026-03-02

**Authors:** Mahmoud Aberkane, Nassira Karich, Chaimae Daoudi, Anass Haloui, Amal Bennani

**Affiliations:** 1 Department of Anatomopathology, Mohammed VI University Hospital Center, Faculty of Medicine and Pharmacy of Oujda, Oujda, MAR

**Keywords:** entrapped respiratory epithelium, hmga2-lpp, multiple myeloma, popcorn, pulmonary hamartoma

## Abstract

Pulmonary hamartoma is a rare benign tumor of the lung. It predominantly affects older males and is frequently peripherally located. On chest radiography, it classically appears as a “coin lesion” exhibiting characteristic popcorn-like calcifications.

We report the case of a 62-year-old male patient, a former smoker, being treated for multiple myeloma. A computed tomography (CT) scan revealed a solid, bilobed pulmonary nodule in the lingula measuring 8 mm in the long axis, for which he underwent surgical resection. Macroscopically, the tumor appeared whitish, multilobulated, well-defined, and firm in consistency. Histologically, the lesion is composed of four components: a predominant cartilaginous component with a lobulated architecture, made up of regular chondrocytes; an adipose component, made up of mature adipocytes; a muscular component made up of smooth, non-atypical muscle fibers; and, focally, a fibromyxoid component, associated with an entrapped ciliated respiratory epithelium. Immunohistochemical staining demonstrated positivity for smooth muscle actin and desmin in the smooth muscle cells.

Through this case, we discuss the clinical, radiological, histopathological, molecular, and therapeutic aspects of pulmonary hamartoma, as well as the main differential diagnosis, based on data from the literature.

## Introduction

The term “hamartoma” was first described by the German pathologist Eugen Albrecht in 1904 [1] and defined as a pseudotumoral malformation characterized by excessive production or abnormal distribution of elements of indigenous tissue, that is, tissue normally belonging to the organ of origin [2].

Initially, hamartomas were thought to derive from embryonic remnants, but this theory was rejected after the discovery of karyotype abnormalities and the presence of recombinations between chromosome bands 6p21 and 14q24. Actually, it is classified as a true benign mesenchymal tumor [3].

Pulmonary hamartoma (PH) is rare. It generally affects middle-aged or elderly adults. Men are more frequently affected than women [4,5]. This tumor grows slowly and is often asymptomatic unless it is located endobronchially, causing obstruction [6]. It is often discovered incidentally and appears on chest X-rays as a coin-shaped lesion with popcorn-like calcification, particularly at the periphery of the lung parenchyma [7]. Cytological or histological examination allows a definitive diagnosis, ruling out differential diagnosis, particularly malignant tumors [6].

We report the case of a patient with multiple myeloma who underwent a follow-up computed tomography (CT) scan that revealed a pulmonary parenchymal lesion in the left lung.

## Case presentation

A 62-year-old patient, a former smoker and alcohol user (sober since 2022), with a history of multiple myeloma since 2022, having received cycles of chemotherapy, was admitted for a pre-bone marrow transplant workup. The clinical examination found the patient conscious and stable on hemodynamic and respiratory levels. The physical examination shows no abnormalities. The patient was afebrile with a preserved general condition.

The laboratory tests showed normal hemoglobin level (13.8 g/dL), platelet count (220000/µL), serum calcium (90 mg/L), total proteins (70 g/L), CRP (1.15 mg/L), and urea (0.32 g/L). Leukocyte count was elevated (17,160/µL), while serum creatinine was slightly increased (12.13 mg/L). Serum protein electrophoresis did not reveal any monoclonal component.

**Figure 1:**
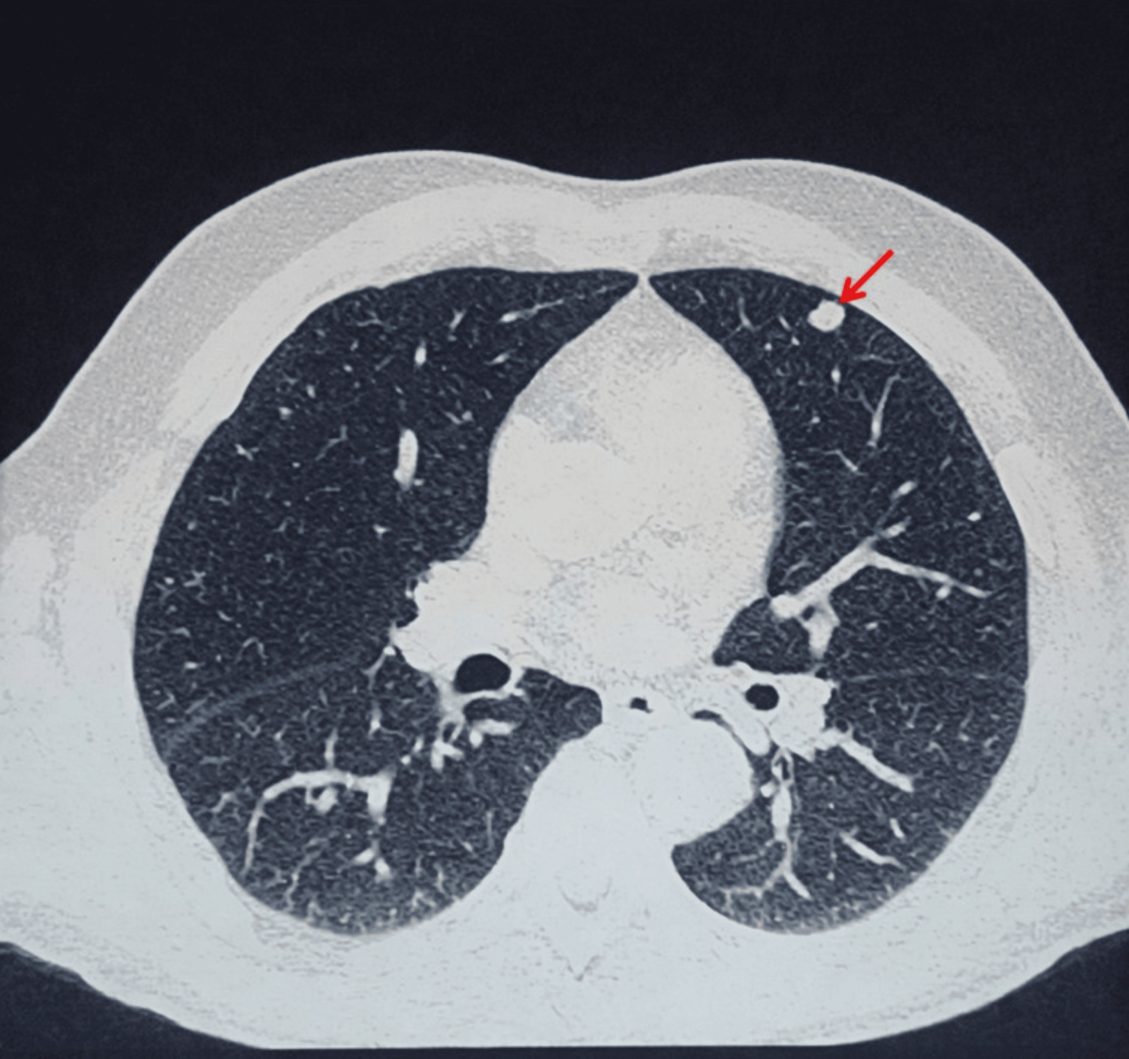
An axial chest CT section demonstrating a well-circumscribed bilobed pulmonary nodule located in the lingula (red arrow), measuring 0.8 x 0.7 cm, in a 62-year-old male

A CT scan of the thorax, abdomen, and pelvis was performed, revealing lytic and microlytic bone lesions in the axial and peripheral skeleton consistent with his pathology, associated with the presence of a solid, bilobed pulmonary nodule in the lingula, measuring 0.8 x 0.7 cm (Figure 1).

A wedge resection of the pulmonary nodule was performed. Macroscopically, the lung resection specimen measures 3.8 x 2.6 x 3.1 cm. On sectioning, a whitish, multilobulated, well-circumscribed nodule of firm consistency is noted, measuring 1.3 x 1.1 x 0.7 cm.

**Figure 2:**
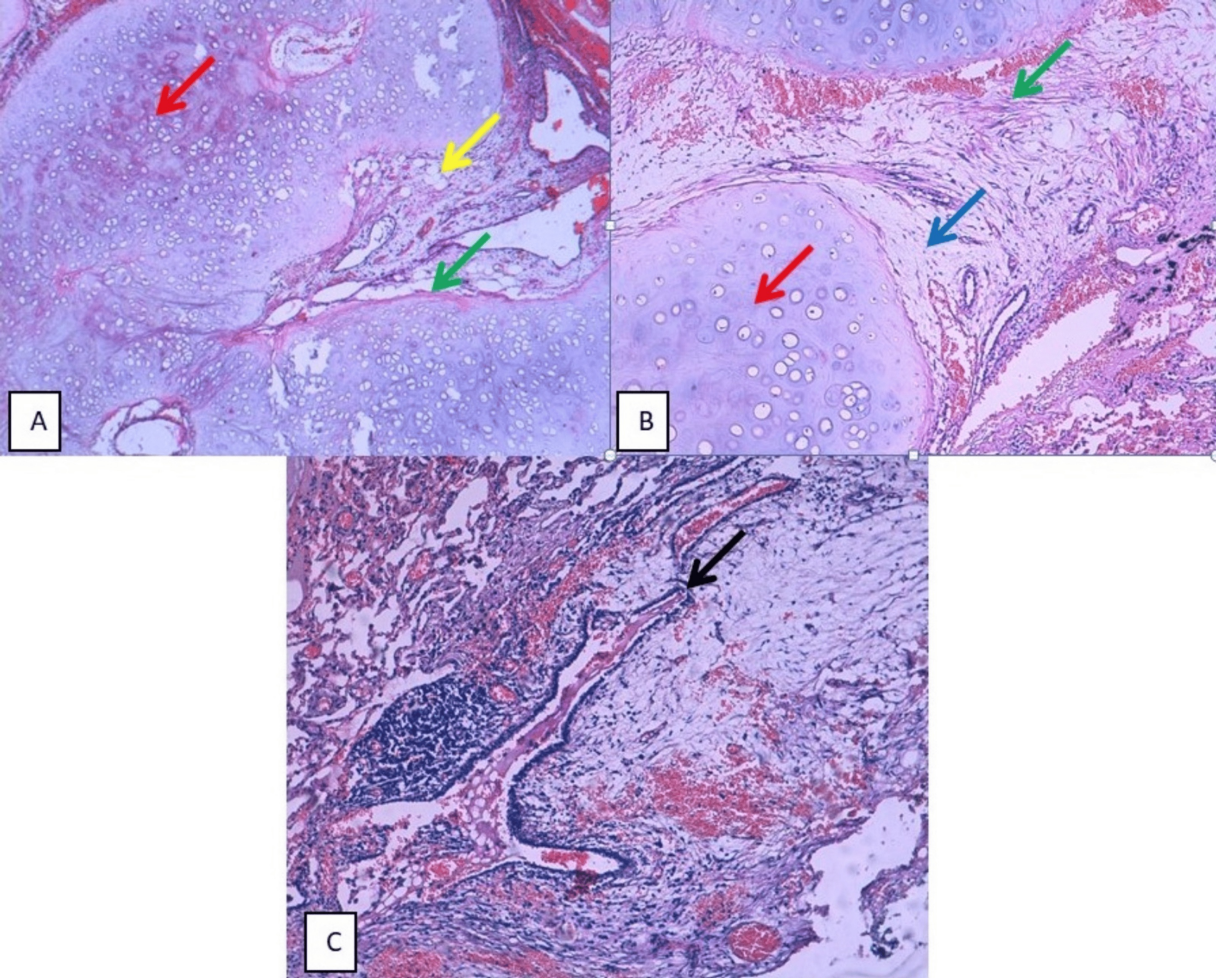
(A) HE-stained histological section showing benign tumor proliferation consisting of a predominant cartilaginous component (red arrow), an adipose component (yellow arrow), and a smooth muscle component (green arrow) (x40). (B) Proliferation of hyaline cartilage tissue (red arrow) associated with a smooth muscle (green arrow) component and fibromyxoid tissue (blue arrow) (x100). (C) Note the presence of entrapped respiratory epithelium within the tumor proliferation (black arrow) (x100).

Histological examination revealed lung parenchyma containing a benign tumor proliferation composed of four components. The first was a predominant cartilaginous component with a lobulated architecture and low cellularity, consisting of regular chondrocytes showing rare binucleation and no evidence of atypia or mitotic activity. The second component was adipose, consisting of cells with clear, vacuolar cytoplasm and peripherally located nuclei. The third component consisted of muscle tissue made of non-atypical smooth muscle fibers (Figure 2, Panel A). Focally, a fourth component consisting of fibromyxoid tissue was noted (Figure 2, Panel B). The proliferation contained entrapped respiratory epithelium, forming characteristic clefts (Figure 2, Panel C).

**Figure 3:**
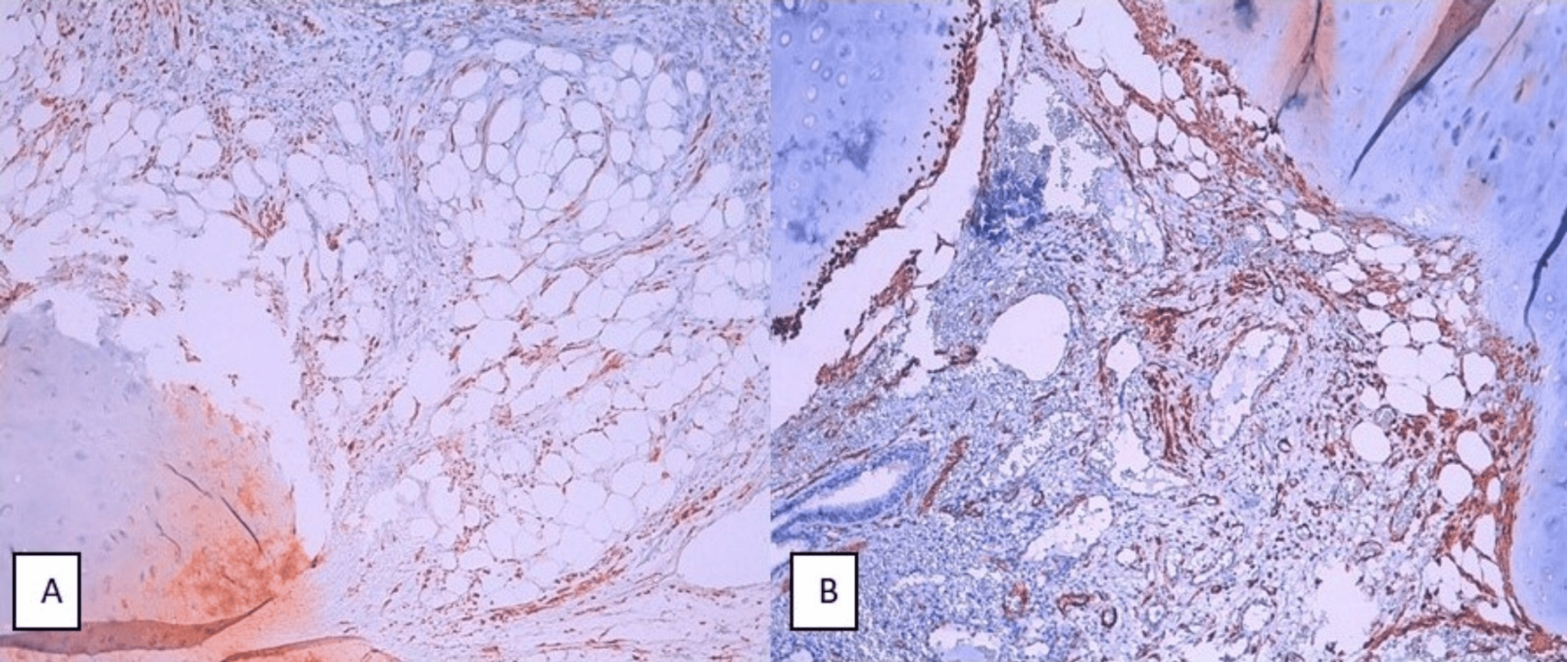
Immunohistochemical staining of smooth muscle cells by desmin (A) and smooth muscle actin (B) (x100)

An immunohistochemical study was performed, showing positive staining of smooth muscle cells by desmin (Figure 3, Panel A) and smooth muscle actin (SMA) (Figure 3, Panel B). These histological and immunohistochemical features are consistent with a PH.

## Discussion

PHs are rare tumors. Their incidence varies from 0.25% to 0.32% in the general population [6]. The peak incidence is between the sixth and seventh decades of life. PH generally affects men more than women [6]. This tumor is more rarely described in the pediatric population [8], and it has never been documented as a congenital lesion [9].

To date, the discovery of a PH in the context of multiple myeloma has never been reported. 

PH can be central or peripheral. Most PHs are solitary, located in the peripheral areas of the lung parenchyma, sometimes in association with small bronchi or bronchioles [8,9]. They are well circumscribed, and their diameter varies from a few millimeters to 20 cm (the largest lesion, according to Tyagi et al., measures 25.5 cm in longest diameter) [6]. The central type of hamartoma, which is rarer, accounts for approximately 10% of cases. It is located in the bronchi and appears as an endobronchial polypoid mass covered by respiratory mucosa [9]. In rare cases, more than 100 nodules may coexist within the same lobe [9].

PH can develop in any lobe of the lung, but it has been particularly reported in the right lower lobe [10]. The clinical manifestations of PH are generally nonspecific. Respiratory infection or pulmonary atelectasis due to mechanical obstruction of the bronchi may be observed [3].

On imaging, hamartoma usually presents as well-defined nodules or masses (generally less than 4 cm in diameter) with lobulated edges [8]. CT imaging is highly effective in detecting intralesional fat or calcifications [11].

The distribution of calcifications within the lesion, giving a “popcorn” appearance, and the presence of fat within the nodule are pathognomonic signs of hamartoma, thereby requiring no further investigation [11]. In a study by Grigoraş et al., calcifications were reported in only 14.81% of cases, which was probably due to the small size of the lesions [12].

Several studies have shown that PHs can be diagnosed in most cases using cytological samples obtained by transthoracic fine needle aspiration biopsy. Its reliability has been demonstrated in cases of pulmonary masses. When indicated, it avoids the need for diagnostic thoracotomy [9].

The material collected by fine needle aspiration is characterized by fibromyxoid stroma, cartilage, bronchial cells, adipose tissue, and, rarely, bone marrow [7]. The identification of fibromyxoid stroma is more reliable than that of cartilage, which is present in only a minority of cases [7]. Bronchial cells with reactive atypia may be a source for false-positive results. Immunohistochemical staining using the S-100 protein can be used to identify chondroid and fibromyxoid stromal cells [7].

Histologically, PHs are composed of a disorganized proliferation of mature mesenchymal tissue [9]. The most typical form contains cartilage but may also contain adipose tissue, bone, smooth muscle, or fibromyxoid tissue in varying proportions and rarely bone marrow [6,9]. As PH expands, the mesenchymal proliferation entraps adjacent airways and creates a cleft-like configuration of respiratory epithelium. This epithelium may be cuboidal or cylindrical and may be the site of metaplasia, hyperplasia, or papillary changes [6,9].

Cases of adenocarcinoma, squamous cell carcinoma, and sarcoma arising from PHs have been reported [1]. However, no pathophysiological link has been established between PHs and the development of lung carcinoma [6]. This association is likely explained by the incidental discovery of asymptomatic hamartomas during diagnostic evaluation for malignant lung tumors [6]. Nevertheless, the risk of lung cancer in patients with PHs has been estimated to be more than six times higher than that observed in the general population [1].

PHs frequently harbor the translocation t(3;12)(q27-28;q14-15), a cytogenetic feature shared with lipomas [13]. At the molecular level, this translocation results in the fusion of the *HMGA2* (high-mobility group AT-hook 2) and *LPP* (LIM domain containing preferred translocation partner) genes [13-15]. The *HMGA2 *gene encodes a member of the high-mobility group (HMG) non-histone chromosomal protein family. HMG proteins function as architectural factors. They contain structural DNA-binding domains and act as transcriptional regulatory factors [15].

The *LPP* gene encodes a member of the LIM domain protein subfamily. The encoded protein is localized to the cell periphery in focal adhesions and may be involved in cell adhesion and cell motility [14]. The *HMGA2-LPP* fusion gene typically consists of exons 1 to 3 of the *HMGA2* gene and exons 9 to 11 of the *LPP* gene [13].

The differential diagnosis of PH includes tumors such as pulmonary chondroma, monomorphic tumors such as lipoma and leiomyoma, and well-differentiated chondrosarcoma [12].

Pulmonary chondromas generally occur in patients with Carney’s triad and consist exclusively of hyaline cartilage, often presenting myxoid changes, surrounded by a thin fibrous pseudocapsule, and notably lacking entrapped respiratory epithelium [16]. Immunohistochemical analysis using SDHB (succinate dehydrogenase B) antibody shows abnormal loss in chondromas associated with Carney syndrome, but not in PH [16].

Pulmonary lipomas are exceedingly rare benign tumors characterized by a proliferation of mature adipocytes beneath the respiratory epithelium; rarely associated with osteocartilaginous metaplasia [12]. These tumors generally lack atypical adipocytes or lipoblasts. On immunohistochemistry, the cells express S100 protein, a standard marker for mature adipocytes [12].

Pulmonary leiomyoma is a rare entity that may present as a primary or metastatic lesion. Primary pulmonary leiomyoma accounts for less than 2% of all benign lung tumors [17]. Histologically, leiomyomas are composed of disorganized fascicles of smooth muscle with minimal vascular or fibrous stroma. Immunohistochemical analysis typically demonstrates strong positivity for smooth muscle markers, including α-SMA (alpha-SMA), desmin, and SMMHC (smooth muscle myosin heavy chain) [17].

Well-differentiated primary pulmonary chondrosarcoma generally presents as an ill-defined lobule containing differentiated hyaline cartilage, composed of cells with plump nuclei, binucleation, hyperchromasia, and coarse chromatin. Myxoid changes in the stroma and necrosis of the chondroid matrix may be observed [18]. Tumor cells are negative for cytokeratins, CD31, and desmin, while S100 protein expression is variable [12,18].

Other differential diagnosis includes benign solid tumors that exhibit ossification or calcification, such as amyloidomas and pulmonary osteomas [8]. Nodular pulmonary amyloidosis is a localized type of amyloid deposition in the lung parenchyma [8]. It appears on CT scan as one or more solid nodules, which may present calcifications [19]. Histologically, nodular amyloidosis is characterized by nodules consisting of dense, eosinophilic material deposits that exhibit apple-green birefringence with Congo red staining under polarized light [8,19].

Pulmonary osteoma is a rare benign bone tumor that appears on CT scan as a lesion with cortical density [8]. Histologically, this lesion consists of mature lamellar bone with Haversian canals. The intratrabecular tissue may contain vascular, fibrous, adipose, and hematopoietic elements [8,20].

Currently, the treatment of conventional PHs is based on surgical resection, which remains the only definitive therapeutic option [10]. Most hamartomas are non-expansive or slow-growing neoplasms; therefore, some authors emphasize the value of surgery only in cases of expansive tumors in young or middle-aged patients or when there are obvious pulmonary symptoms [3].

According to some authors, PHs can increase in size, recur, or contribute to malignant transformation through chronic focal inflammation [3]. Therefore, surgical resection is indicated in cases of isolated lung tumors measuring more than 2.5 cm or when the possibility of a malignant tumor cannot be ruled out [3].

Technically, the main objective of the surgery is to preserve functional lung tissue. Therefore, enucleation and wedge resection are the most commonly used surgical techniques [10]. However, a lobectomy and, rarely, a pneumonectomy are recommended in cases of large, multiple, or centrally located lesions, or when wedge resection is not possible [10].

Due to the potential risk of recurrence, it should be emphasized that patients presenting with hamartomas require a complete evaluation and regular follow-up [3].

## Conclusions

PH is a rare benign tumor of the lung. Often asymptomatic, it is usually discovered incidentally on chest X-ray in the form of a coin-shaped lesion with popcorn-like calcification. Histological examination confirms the diagnosis by the presence of an abnormal mixture of mature mesenchymal tissues associated with entrapped respiratory epithelium. Several studies have indicated that the incidence of lung cancer is higher in cases of pulmonary hamartoma, but no pathophysiological link between the two lesions has been established. Histological examination is useful for ruling out differential diagnosis of pulmonary hamartoma and is generally sufficient.

Furthermore, identification of the *HMGA2-LPP* fusion gene, resulting from the t(3;12)(q27-28;q14-15) translocation, further facilitates the diagnosis of pulmonary hamartoma. Surgery remains the only curative treatment, particularly in symptomatic cases or when malignancy cannot be definitively excluded.
